# Supplementary material for: Programmable multimodal actuation in cholesteric liquid crystal elastomer hollow fibers beyond mechanochromism
Source: Nat Commun. 2026 Mar 27;17:4510. doi: 10.1038/s41467-026-71050-6 (PMC13187455; doi:10.1038/s41467-026-71050-6)
Supplement: Supplementary file 1 — Supplementary Information [file 41467_2026_71050_MOESM1_ESM.pdf]

# Supplementary Information

## Programmable multimodal actuation in cholesteric liquid crystal elastomer hollow fibers beyond mechanochromism

*Jiazhe Ma*[1], *John S. Biggins**[2], *Fan Feng**[3] and *Zhongqiang Yang**[1,4]

[1]Key Lab of Organic Optoelectronics and Molecular Engineering of Ministry of Education, Department of Chemistry, Tsinghua University, Beijing 100084, P. R. China

[2]Department of Engineering, University of Cambridge, Trumpington St., Cambridge CB2 1PZ, UK

[3]School of Mechanics and Engineering Science, Peking University, Beijing 100871, P. R. China

[4]Laboratory of Flexible Electronics Technology, Tsinghua University, Beijing 100084, P. R. China

E-mail: jsb56@cam.ac.uk; fanfeng@pku.edu.cn; zyang@tsinghua.edu.cn

**Supplementary Figures:**

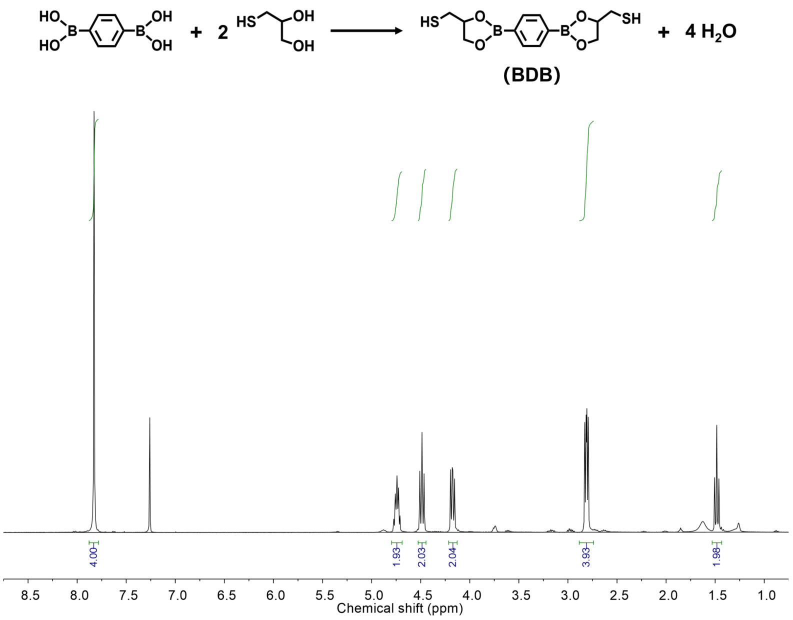


**Supplementary Fig. 1 Reaction scheme and $^1$H NMR spectrum of BDB.** $^1$H NMR (400 MHz, $CDCl_3$): δ 7.83 (s, 4H), 4.80 – 4.69 (m, 2H), 4.49 (t, J = 8.6 Hz, 2H), 4.18 (dd, J = 9.1, 6.6 Hz, 2H), 2.81 (dd, J = 8.6, 5.4 Hz, 4H), 1.48 (t, J = 8.7 Hz, 2H). These data match those previously described in the literature[1].

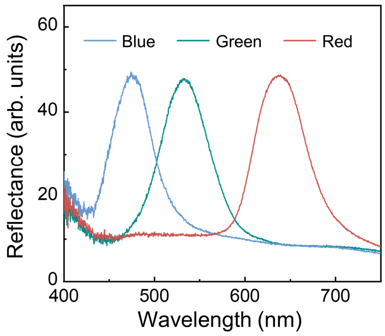


**Supplementary Fig. 2 Reflectance spectra of three CLCE hollow fibers that show red, green and blue.**

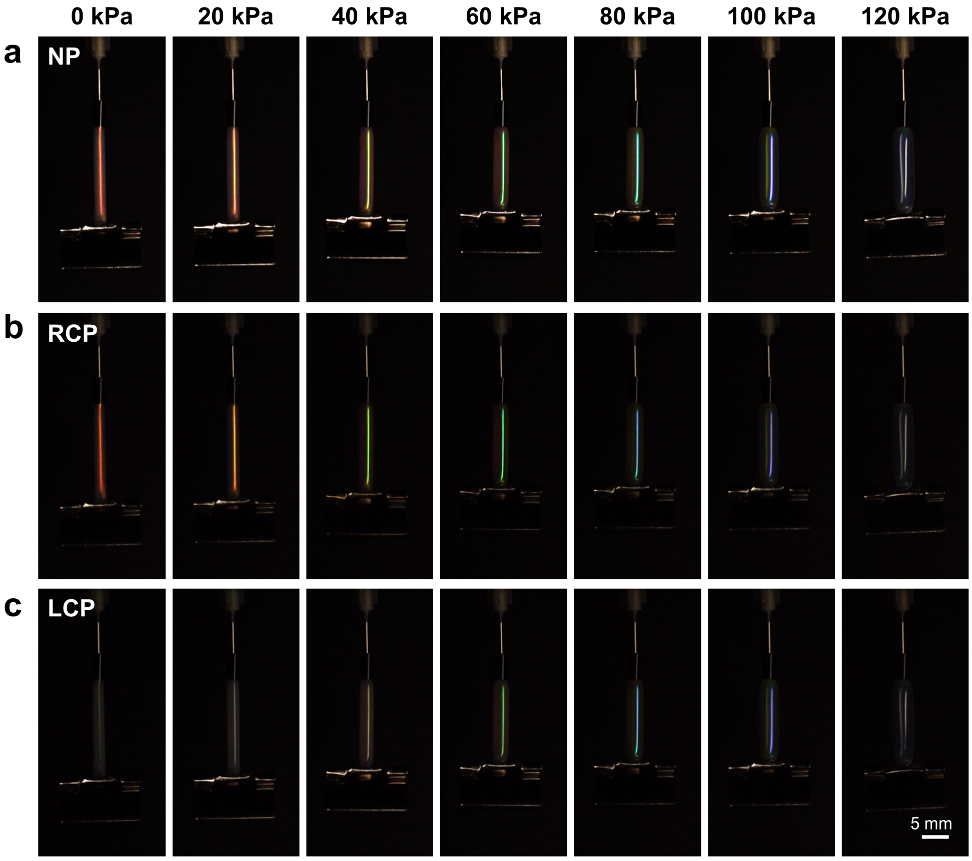


**Supplementary Fig. 3 Images of a red polydomain CLCE hollow fiber inflated at different pressures.** (a) With no polarizer (NP). (b) With right-handed circular polarizer (RCP). (c) With left-handed circular polarizer (LCP).

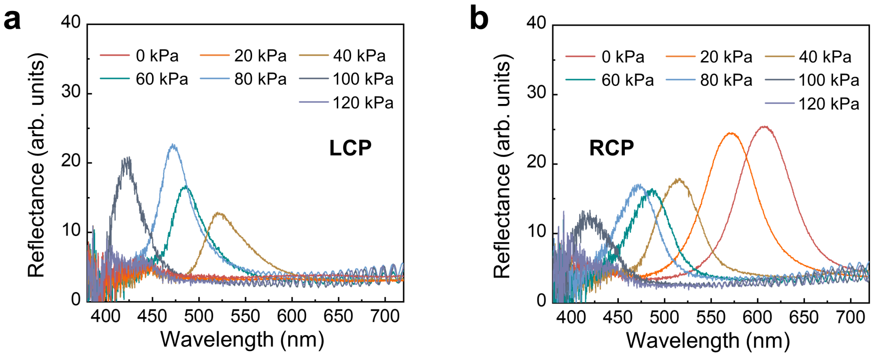


**Supplementary Fig. 4 Reflection spectra of the red polydomain CLCE hollow fiber inflated at different pressures.** (a) Under LCP light. (b) Under RCP light.

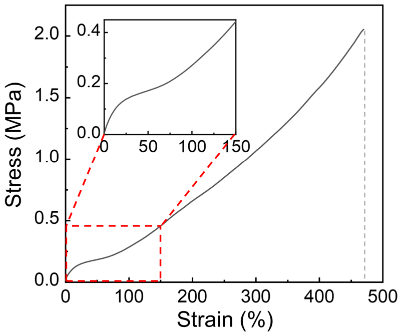


**Supplementary Fig. 5 The stress-strain curve of the polydomain CLCE sample.** The fracture strain was around 470%, with a corresponding stress of 2.0 MPa. The calculated Young's modulus was 0.86 MPa.

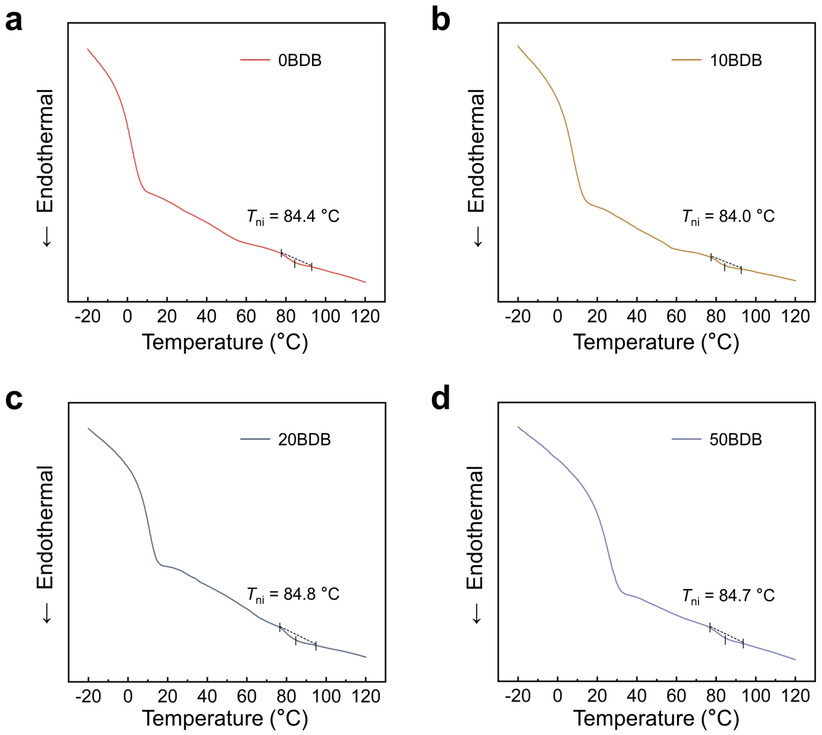


**Supplementary Fig. 6 Differential scanning calorimetry characterization of CLCE hollow fibers with different molar ratios of BDB.** (a) 0BDB. (b) 10BDB. (c) 20BDB. (d) 50BDB. The arrows indicate the endothermic direction.

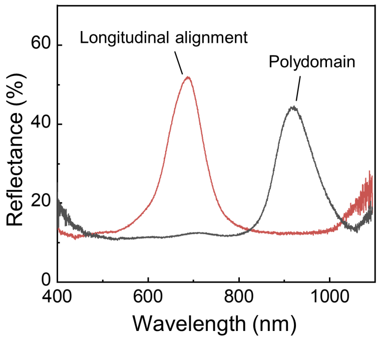


**Supplementary Fig. 7 Reflectance spectra of the polydomain sample before programming and the longitudinally aligned sample after programming during the fabrication of longitudinally aligned CLCE hollow fibers.** It is worth noting that although a dark reference was calibrated during testing, the inherent thickness (diameter) of the sample inevitably placed it closer to the light source than the dark reference, resulting in an increased baseline value in the curves. The fiber reflectivity is not the peak value of the curve but the difference between the peak value and the baseline. Through Lorentz fitting, the reflectivity of the fiber before and after programming was calculated to be 39% and 44%, respectively. This indicates that although stretching distorted the helical axis of the CLCE, its reflective performance was not weakened. Instead, it may have increased due to the simultaneous reflection of both left- and right-handed circularly polarized light.

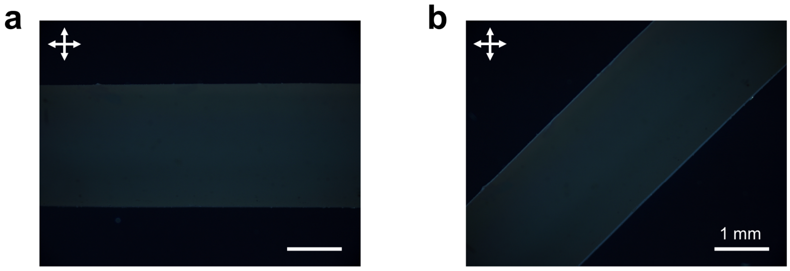


**Supplementary Fig. 8 Polarized optical microscopy (POM) of the longitudinally aligned CLCE hollow fiber before programming.** (a) The fiber axis was aligned parallel to the polarizer. (b) The fiber axis was aligned at 45° to the polarizer.

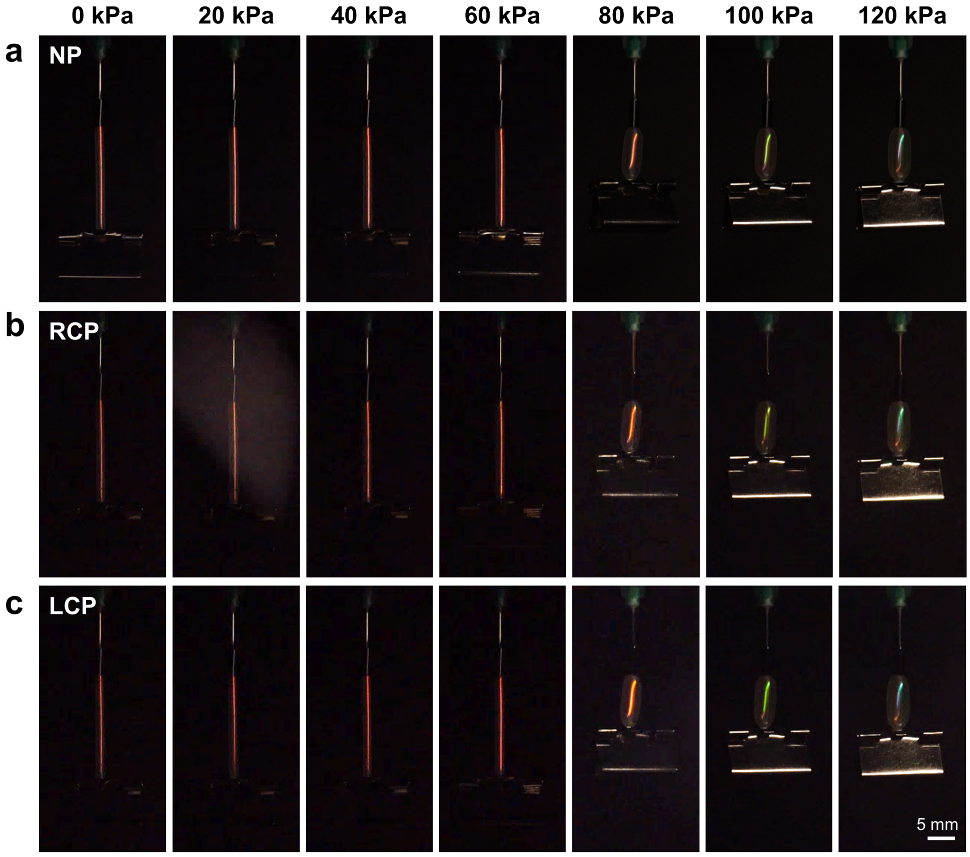


**Supplementary Fig. 9 Images of a red CLCE hollow fiber with longitudinal alignment inflated at different pressures.** (a) With NP. (b) With RCP. (c) With LCP.

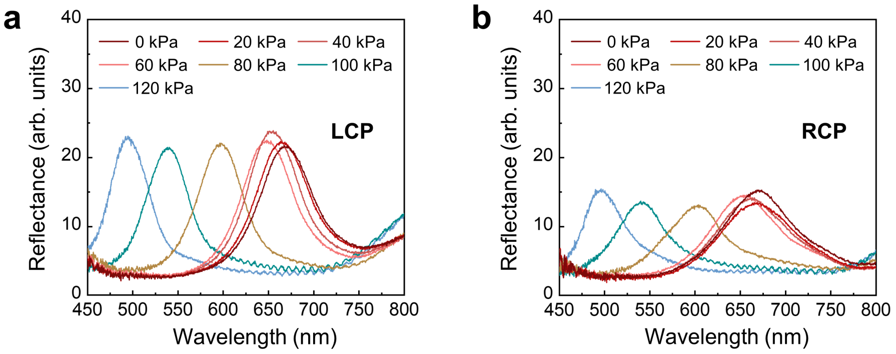


**Supplementary Fig. 10 Reflection spectra of the red CLCE hollow fiber with longitudinal alignment inflated at different pressures.** (a) Under LCP light. (b) Under RCP light.

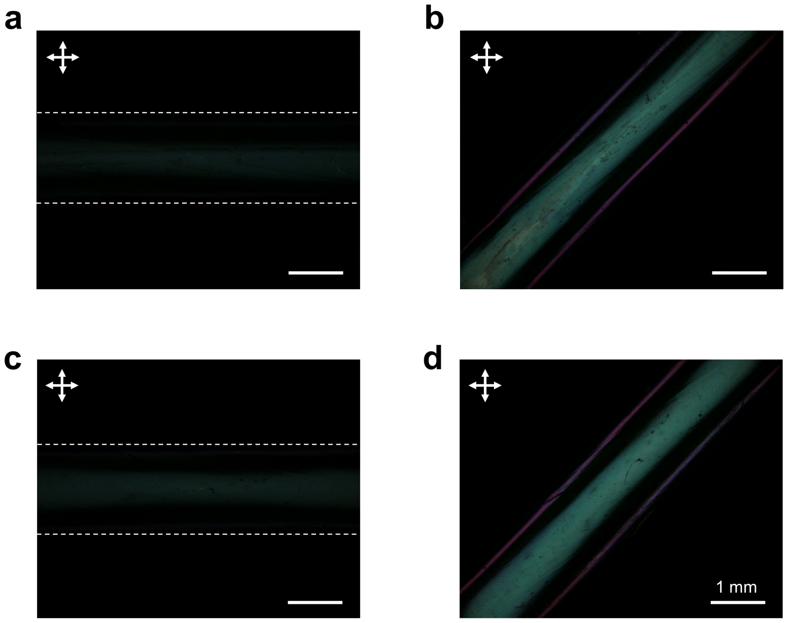


**Supplementary Fig. 11 POM images of twisted CLCE hollow fibers with different twist densities.** (a and b) Fibers with a right-handed twist of 9° $mm^{-1}$, with the fiber axis oriented (a) parallel to the polarizer and (b) at 45° to the polarizer. (c and d) Fibers with a right-handed twist of 18° $mm^{-1}$, with the fiber axis oriented (c) parallel to the polarizer and (d) at 45° to the polarizer.

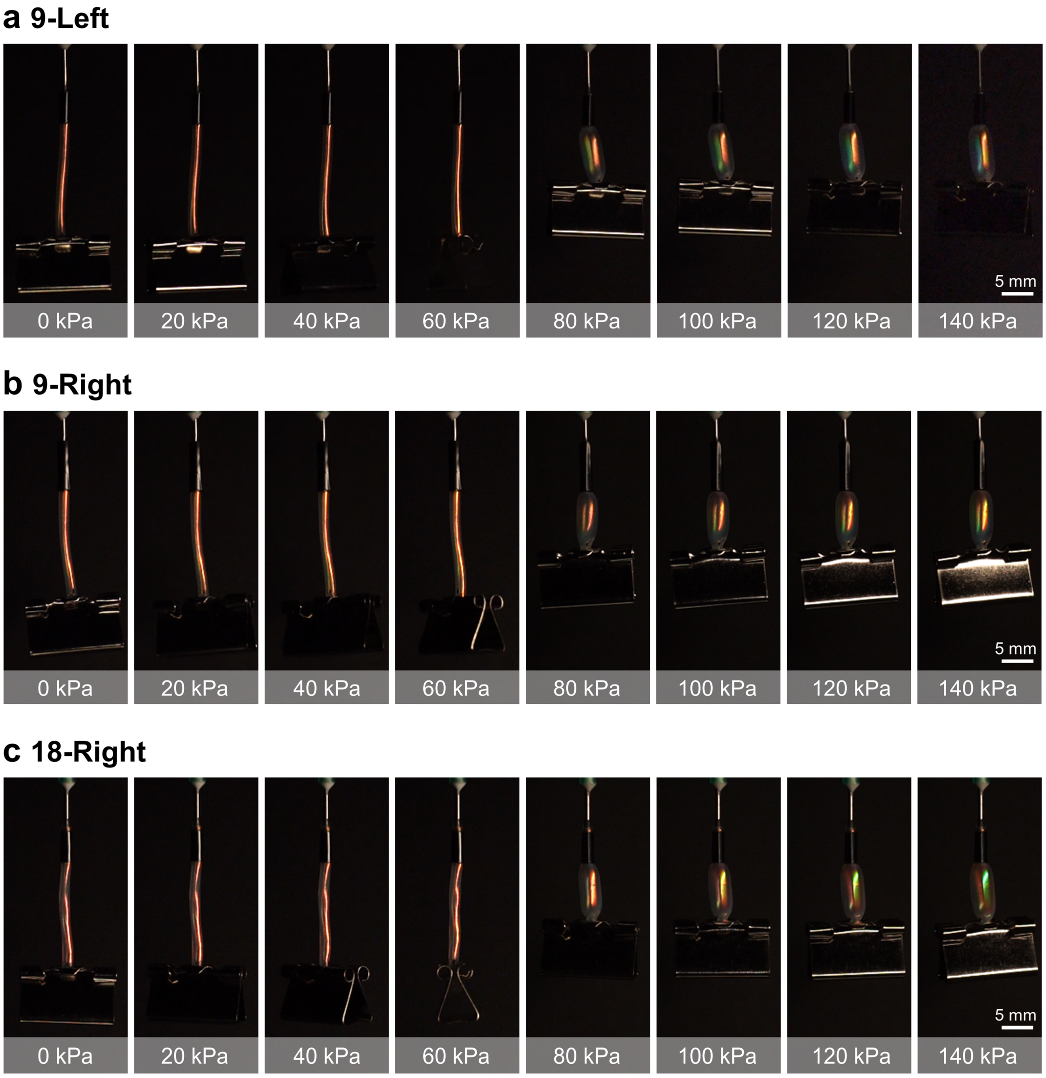


**Supplementary Fig. 12 Pneumatic response of twisted CLCE hollow fibers with different twist densities and handedness.** (a) 9° $mm^{-1}$ left-handed twist. (b) 9° $mm^{-1}$ right-handed twist. (c) 18° $mm^{-1}$ right-handed twist.

**Supplementary References:**


1 Chen, Y. *et al.* Covalently cross-linked elastomers with self-healing and malleable abilities enabled by boronic ester bonds. *ACS Appl. Mater. Interfaces* **10**, 24224-24231 (2018).
