# Supplementary material for: Programmable multimodal actuation in cholesteric liquid crystal elastomer hollow fibers beyond mechanochromism
Source: Nat Commun. 2026 Mar 27;17:4510. doi: 10.1038/s41467-026-71050-6 (PMC13187455; doi:10.1038/s41467-026-71050-6)
Supplement: Supplementary file 2 — Description of Additional Supplementary Files [file 41467_2026_71050_MOESM2_ESM.pdf]

Description of Additional Supplementary Files

File name: Supplementary Movie 1

Description: Pneumatic response of a polydomain CLCE hollow fiber. The air pressure was set directly to 100 kPa, and the valve was opened for rapid inflation.

File name: Supplementary Movie 2

Description: Pneumatic response of a longitudinally aligned CLCE hollow fiber. The air pressure was first set to 80 kPa, and the valve was opened to begin inflation. Once the shape and color of the fiber no longer changed, the air pressure was then increased to 120 kPa for further inflation.

File name: Supplementary Movie 3

Description: Pneumatic response of a circumferentially aligned CLCE hollow fiber. The air pressure was set directly to 200 kPa, and the valve was opened for rapid inflation.

File name: Supplementary Movie 4

Description: Pneumatic response of a CLCE hollow fiber with 18° mm^{-1} left-handed twist. The air pressure was first set to 80 kPa, and the valve was opened to begin inflation. Once the shape and color of the fiber no longer changed, the air pressure was then increased to 140 kPa for further inflation.
